# Gender differences in the association of dementia and depression

**DOI:** 10.1192/j.eurpsy.2024.1328

**Published:** 2024-08-27

**Authors:** R. Fernández Fernández, P. del Sol Calderón, Á. Izquierdo de la Puente

**Affiliations:** ^1^Psychiatry, Hospital Universitario Infanta Cristina, Parla; ^2^Psychiatry, Hospital Universitario Puerta de Hierro Majadahonda, Majadahonda, Spain

## Abstract

**Introduction:**

The evolution of depression and dementia has been shown to differ in some studies. For example, a history of recent depression has been found to be associated with an increased risk of Alzheimer’s disease in women (Kim et al., 2021).

**Objectives:**

We will use data collected from several dementia studies to analyze whether the presence of depression at diagnosis is more frequent in women.

**Methods:**

We conducted a systematic search for articles analyzing the presence of depression in patients with a diagnosis of dementia. We analyzed by Student’s t test the presence of depression according to sex, considering the alternative hypothesis that there is more depression in female than male patients.

**Results:**

The mean age of the sample was 71 years. We obtained a statistically significant Student’s t test (p=0.02).

**Conclusions:**

The approach and approach to depression in the elderly as a risk factor could be different according to sex. For example, some studies have proposed the use of hormone replacement therapy (HRT) after menopause as a possible protective factor for the subsequent development of dementia (Kim et al., 2022). Further studies are recommended in this regard.

**Disclosure of Interest:**

None Declared

